# *FLC* expression is down-regulated by cold treatment in *Diplotaxis tenuifolia* (wild rocket), but flowering time is unaffected

**DOI:** 10.1016/j.jplph.2017.03.015

**Published:** 2017-07

**Authors:** Jemma L. Taylor, Andrea Massiah, Sue Kennedy, Yiguo Hong, Stephen D. Jackson

**Affiliations:** a School of Life Sciences, University of Warwick, Coventry CV4 7AL, UK; b Elsoms Seeds Ltd, Pinchbeck Road, Spalding PE11 1QG, UK; c Research Centre for Plant RNA Signaling, College of Life and Environmental Sciences, Hangzhou Normal University, Hangzhou 310036, China

**Keywords:** Bolting time, *Diplotaxis tenuifolia*, Photoperiod, Vernalization, Wild rocket

## Abstract

Wild rocket (*Diplotaxis tenuifolia*) has become a very popular salad leaf due to its peppery taste. It is part of the *Brassicaceae* family and thus has a high level of homology at the DNA level to other Brassica species including *Arabidopsis thaliana*. The vernalization and photoperiodic requirements of wild rocket have not been reported to date. Photoperiodic experiments described here demonstrate that rocket is a facultative long day plant. To investigate the vernalization requirement, both seed and young plants were given vernalization treatments at 4 °C for different lengths of time. A rocket homologue of *FLOWERING LOCUS C* (*DtFLC*) was isolated and shown to functionally complement the *Arabidopsis* FRI*^+^flc3* null mutant. Whilst the expression of *DtFLC* was significantly reduced after just one week of cold treatment, cold treatments of two to eight weeks had no significant effect on bolting time of wild rocket indicating that rocket does not have a vernalization requirement. These findings illustrate that important fundamental differences can exist between model and crop plant species, such as in this case where down-regulation of *DtFLC* expression does not enable earlier flowering in wild rocket as it does in *Arabidopsis* and many other Brassica species.

## 1 Introduction

**Fig. 1:**
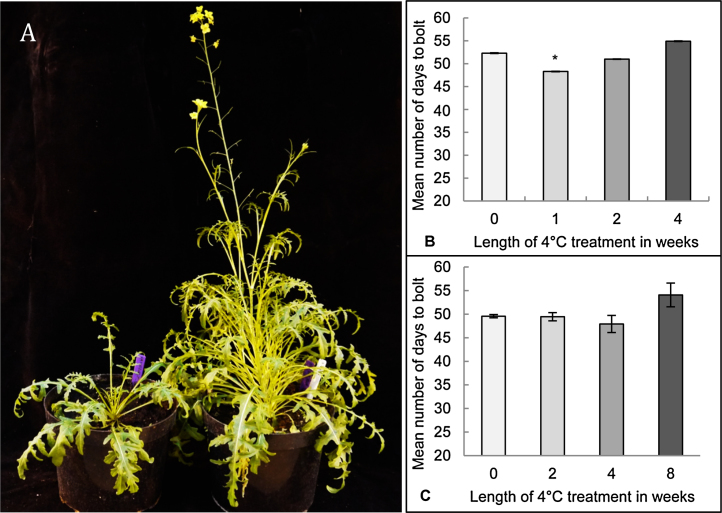
Effect of vernalization at 4 °C on D.tenuifolia. A) Photo of a non-bolting rocket plant (left) and one that has bolted and flowered (right). B) The mean number of days to bolt of plants where the seed was subjected to cold treatment at 4 °C for one, two or four weeks. Bars show mean ± SE (n = 14 (0 weeks), n = 15 (1 week and 2 weeks), n = 10 (4 weeks)). Student’s *t*-test was used to compare the number of days to bolt of each vernalization treatment (one, two and four weeks) against the ambient conditions (0 weeks). *Statistical significance of p < 0.05 C) Mean number of days to bolt for 4 week old plants subjected to cold treatment at 4 °C for two, four and eight weeks. The length of the vernalization treatment was subtracted from the number of days to bolt. Bars show mean ± SE (n = 14 (0 weeks), n = 15 (2 weeks and 4 weeks), n = 13 (8 weeks)). Student’s *t*-test was used to compare the number of days to bolt of each vernalization treatment (2, 4 and 8 weeks) against the ambient conditions (0 weeks). There was no significant difference in number of days to bolt between vernalization treatments and ambient conditions (p < 0.05)

Wild rocket (*Diplotaxis tenuifolia*) has increased in popularity over the last 20 years in the leafy salads market (Chun et al., 2013), in the UK alone over 80 tons of rocket is consumed per week (Gill, 2008). The genus *Diplotaxis* is found in the *Brassicaceae* family in the *Oleracea* clade (Arias and Pires, 2012) and is therefore closely related to species *Brassica rapa*, *Brassica juncea*, *Brassica napus* and *Brassica oleracea,* as well as *Arabidopsis thaliana* (Arabidopsis). Flowering is undesirable in commercial rocket production as pre-harvest flowering can lead to the crop being unsaleable (Fig. 1a), despite this very little work has been reported on the control of flowering in wild rocket. Here we investigate the vernalization and photoperiodic requirements of this relatively new salad crop species.

Regulation of flowering time involves a complex gene network which allows a plant to respond to both internal and external signals in order to control the timing of the transition from the vegetative phase to the reproductive phase (Srikanth and Schmid, 2011). The timing of this transition is crucial to enable reproduction to occur when the plant is at its fittest and the environmental conditions are most favorable (Thomas et al., 2006). Most research into flowering time has been conducted in the model plant Arabidopsis through the generation and testing of many mutant and transgenic overexpression lines to elucidate gene function (Corbesier and Coupland, 2005). Using the knowledge gained from Arabidopsis and applying it to crop species is helping our understanding of the control of flowering in crop plants. Currently there are six key pathways that regulate flowering time: the photoperiodic, autonomous, vernalization, gibberellic acid, age dependent and ambient temperature pathways (Brambilla and Fornara, 2013, Fornara et al., 2010, Jarillo and Pineiro, 2011, Srikanth and Schmid, 2011). All of these pathways converge on the floral pathway integrator genes such as *FLOWERING LOCUS T* and *SUPPRESSOR OF OVEREXPRESSION OF CONSTANS 1* which activate the downstream floral meristem identity genes. When flowering is induced the shoot apical meristem changes from forming vegetative tissues such as leaves to form flowers.

Vernalization is the attainment or acceleration of floral competence as a consequence of prolonged exposure to cold temperature (Chouard, 1960). In vernalization-responsive plant species, vernalization conveys the ability of the plant to flower and can greatly reduce the time it takes to flower (Guo et al., 2004, Hackett and Hartmann, 1967, Jung and Mueller, 2009, Strange et al., 2011). The cold treatment causes epigenetic changes that reduce levels of a floral repressor gene *FLOWERING LOCUS C* (*FLC*). When the plant is returned to a higher temperature these epigenetic changes are stable enough to maintain the repression of *FLC* expression and the reduced levels of this repressor thus enables flowering to be induced (Boss et al., 2004). Studies of Arabidopsis ecotypes show that there are differences in the requirement for vernalization. Rapid cycling ecotypes will flower without the need for a vernalization treatment, whereas winter annual ecotypes will flower very late unless exposed to a period of vernalization (Nordborg and Bergelson, 1999, Wang, 2014). It was found that these vernalization response phenotypes all relate to the levels of expression of *FLC* (Song et al., 2012). Plants which have a requirement for vernalization tend to be those from seasonal climates where adaptation to extended periods of cold can be advantageous. The stage in the plant’s lifecycle which is responsive to vernalization varies between species as does the length of treatment needed in order to fully satisfy the vernalization requirement. Arabidopsis is able to respond to vernalization as both seed and young plants (Nordborg and Bergelson, 1999, Strange et al., 2011), whereas many *Brassica* species only respond to a vernalization treatment as young plants (Jung and Mueller, 2009). Rocket is closely related to both Arabidopsis and *Brassica* and it is not currently known at which stage of its lifecycle it is able to respond, what the optimum vernalization period might be, or even if it has a vernalization response at all.

The ability of a plant to measure and respond to the length of day, or photoperiod, is often critical to its growth and development, particularly in the timing of the floral transition. Most plants fall into one of three main photoperiodic categories; long day (LD) plants which flower when the photoperiod is longer than a critical length, short day (SD) plants which flower when the photoperiod is shorter than a critical length, and day neutral plants which flower regardless of the photoperiod (Matsoukas et al., 2012). LD plants flower quite quickly in LD, but if subjected to SD conditions they will flower much more slowly (facultative response) or not at all (obligate response), the opposite being true for SD plants.

Understanding the vernalization and photoperiod requirements of commercial crops, and the underlying genes that control these responses in these crops, is of huge benefit to crop breeding programs aiming to create varieties that can be grown successfully in a range of latitudes and climatic conditions. In this paper, we investigate the photoperiodic and vernalization responses of *D.tenuifolia*. We show that wild rocket is a facultative long day plant and that it does not respond to vernalization treatments of several weeks duration even though the expression of the rocket *FLC* orthologue is strongly reduced by these treatments.

## 2 Materials and methods

### 2.1 Plant material

Elsoms Seeds Ltd provided seed of *Diplotaxis tenuifolia* bred through four generations of single seed descent for uniformity of leaf shape, color, and bolting time. *Arabidopsis thaliana* mutant FRI*^+^flc3* null (Col0 background) seed was originally sourced from NASC (http://arabidopsis.info/) and used in the complementation experiments.

### 2.2 DNA and RNA extraction

Rocket genomic DNA was extracted using CTAB extraction method adapted from Stewart and Via (1993) on frozen leaf material, which was ground to a powder using a Dremel Drill with a 1.5 ml Eppendorf tube drill bit. 300 μl CTAB B buffer (100 mM Tris/Cl pH 8.0, 1.4 M NaCl, 20 mM EDTA, 2% hexadecyltrimethyl ammoniumbromide) was added and homogenized. Samples were incubated at 65 °C for 30 min and centrifuged. The supernatant was removed into a new tube and twice extracted using 300 μl Chloroform:Isoamyl alcohol (24:1) and centrifugation. The aqueous top phase was transferred each time. 300 μl CTAB C buffer (1% hexadecyltrimethyl ammoniumbromide, 10 mM EDTA, 50 mM Tris/Cl pH 8.0) was added and left overnight at room temperature. On day two, the tubes were centrifuged and the pellet dissolved in 400 μl 1 M CsCl. DNA is then precipitated using 100% Ethanol and centrifuged. The pellet was then washed twice using 70% Ethanol before drying and resuspending in 25 μl TE buffer (pH 8) with RNase A (Invitrogen) (20 μg/ml).

RNA extraction was done using the Z6 extraction buffer (containing 8 M guanidine hydrochloride) method (Logemann et al., 1987). Frozen leaf, or imbibed seed, samples were ground to a powder using liquid N_2_ and a pestle and mortar. The powder was transferred to a 1.5 ml Eppendorf tube and Z6 buffer plus 2-mecaptoethanol (50 mM final concentration) added. This was homogenized using a Dremel Drill with a 1.5 ml Eppendorf tube drill bit before the rest of the published method was followed. 5 μg RNA was DNase treated using Ambion^®^ Turbo DNA-*free*™ DNase and then resuspended in 12 μl DEPC treated H_2_O. 1 μg of DNase treated RNA was synthesized into cDNA using an Invitrogen Thermoscript cDNA synthesis kit or BioRad iScript cDNA synthesis kit. Arabidopsis RNA extraction was performed using the same method, but with a reduction in the volume of reagents used due to the smaller amount of starting leaf material.

### 2.3 Vernalization experiments

*D. tenuifolia* seed was sown onto damp paper towels and covered with aluminum foil and placed at 4 °C for one, two or four weeks. At the end of the vernalization period, seeds were removed from the paper and put onto F2S soil (Levington) in P40 trays at one seed per cell and placed in the glasshouse at 20 °C with a 16 h photoperiod. The germination date was recorded. Control seed to stay at ambient temperature was sown in the same way but was placed into the glasshouse at 20 °C for one week by which time the seeds had germinated. These were then transferred to F2S soil in P40 trays at one seed per cell. At four weeks post germination, plants were transplanted into 5 inch pots of M2 soil (Levington) to continue growing. In the plant experiment, seed was sown directly to F2S soil (Levington) in P40 cells and put in a controlled environment cabinet (MLR-352, Panasonic Co. Ltd) at 20 °C to aid germination with 16 h photoperiod for five days (light level 104 μmol m^−2^ s^−1^). The germination date was recorded and plants grown until three weeks old in a glasshouse at 20 °C with 16 h photoperiod. These were then transplanted to 5 inch pots of M2 soil (Levington). After one week (four weeks after germination), the plants were moved to 4 °C with 16 h photoperiod to vernalize for two, four or eight weeks. The plants were then returned to glasshouse conditions. Control plants were kept at 20 °C with 16 h photoperiod (ambient conditions) for the entirety of the experiment. Plant material was harvested at the end of each week at 9 h after lights on. The number of days from germination to bolting was recorded, bolting was taken as the point when the height of the bolt was about 10 cm and is the measurement normally used by growers and industry to tell whether a plant has bolted. As the plants were grown in controlled environment most plants in a given treatment bolted around the same time. It is known that in Arabidopsis *FLC* is expressed in seed, shoot and root tissue (Chiang et al., 2009, Michaels and Amasino, 1999) so for analysis of FLC expression all material above soil level was sampled from up to six plants for the seed vernaliszation experiment and for weeks 1–3 of the plant experiment,. From week 4 onwards of the plant vernalization experiment when the plants were larger newly expanded leaves were collected from five plants and pooled together.

### 2.4 Photoperiod experiments

Rocket seeds were sown onto F2S soil (Levington) in p24 trays and topped with vermiculite. These were placed into a controlled environment cabinet (MLR-352, Panasonic Co. Ltd) at 22 °C with 16 h light, 8 h dark. The germination date was recorded and at 10 days after sowing, plants were moved into the different photoperiods. These were short day conditions with 8 h light and 16 h dark, intermediate day conditions with 12 h light and 12 h dark, and long day conditions with 16 h light and 8 h dark. Plants were grown in the controlled environment cabinets under fluorescent light (light levels 124 μmol m^−2^ s^−1^). Plants were transplanted at three weeks from sowing into 5 inch pots of M2 soil (Levington) and returned to the correct photoperiod and time to bolting was recorded for each plant. A second experiment was carried out using incandescent bulbs (Bell Striplite, 30 W, light levels 33 μmol m^−2^ s^−1^) to provide the day extension lighting. The experimental design was as described above but with short day (8:16) conditions with 8 h fluorescent bulb light and 16 h dark, intermediate day conditions (12:12) with 8 h fluorescent bulb light + 4 h incandescent bulb day extension and 12 h dark, and long day conditions (16:8) with 8 h fluorescent bulb light +8 h incandescent bulb day extension and 8 h dark.

### 2.5 Isolation of the *DtFLC* gene from *D. tenuifolia*

Degenerate primers for *FLC* were designed using aligned sequences for *Arabidopsis* and *Brassica FLC* genes and used to PCR amplify *DtFLC* using *D. tenuifolia* cDNA as the template. Specific *DtFLC* primers (Supplementary data Table 1A) were then designed to isolate the remaining sequence using RLM RACE (Invitrogen). 5′ and 3′ UTR primers specific to *DtFLC* (see supplementary data for sequences) were used to amplify the gene from rocket genomic DNA and cDNA. The gene sequence data has been submitted to the GenBank database under accession number KX148480.

### 2.6 Functional complementation in *Arabidopsis*

The *DtFLC* coding sequence was isolated from cDNA using primers which incorporated the start and stop codons. This was cloned into pGEM-T easy and amplified out using GATEWAY™ (Invitrogen) adapter primers specific to *DtFLC* (see supplementary data for sequences). Using BP clonase (Invitrogen), this was cloned into pDONR 207 and then pB2GW7 using LR clonase (Invitrogen). The sequence was verified before *DtFLC* containing pB2GW7 plasmid DNA was transformed into *Agrobacterium tumefaciens* strain c58pGV3101. 500 ml LB (10:5:5) plus 25 μg/ml Gentamycin, 100 μg/ml Spectinomycin and 12.5 μg/ml Rifampicin was inoculated using 5 ml cell culture of *A. tumefaciens* strain c58pGV3101 containing the *DtFLC*-pB2GW7 plasmid. This was incubated at 28 °C for 16 h. The culture was centrifuged and the supernatant removed. 500 ml 5% (w/v) sucrose solution was used to resuspend the cells and 100 μl silwet L-77 added before dipping the inflorescences of the Arabidopsis FRI*^+^flc3* null plants (Clough and Bent, 1998). The plants were sealed in a bag for 24 h before putting at 22 °C with 16 h light, 8 h dark in the glasshouse facility. T_1_ seed was harvested and sown onto soil (Levington F2S:sand:vermiculite fine grade 6:1:1). BASTA (Ammonium glyfosinate (150 g/L)) soil soaking was used at 1:1000 as the selection method. The first treatment was given and the trays were covered and placed at 4 °C for three days. These were removed and put under a propagator lid in a 16 h photoperiod at 22 °C. Four further treatments were done before transplanting. Transformed plants were transplanted into individual pots and leaf number and number of days to bolt were recorded when the primary bolt was 1 cm. Seed was collected from plants showing the expected complementation phenotype and sown onto soil. The pots were stratified at 4 °C for 3 days before being placed at 22 °C with 16 h photoperiod in controlled environment cabinets (MLR-352, Panasonic Co. Ltd). The number of days to flower and rosette leaf number at 1 cm bolt were recorded. Leaf material was collected and RNA extracted as outlined above.

### 2.7 Real time RT-PCR

Real time RT-PCR was performed on samples from plants during the rocket vernalization experiments and also on the Arabidopsis *DtFLC* complementation T_2_ plants. To measure the expression of *DtFLC* in rocket from the vernalization experiments, a mixture of RNA from samples where *DtFLC* expression was likely to be highest, were combined to synthesize cDNA to make the standard for the real time reactions. The standard was diluted 10-fold from 10^0^–10^−4^ to make the standard curve. All real time RT-PCR carried out for the vernalization samples used *DtTIP41*, *DtCACS* and *Dtα-tubulin* as housekeeping genes for normalization (Supplementary data Table 2A). To measure the expression of the *DtFLC* gene in Arabidopsis FRI + *flc3* null plants, *AtActin*, *AtTIP41* and *Atβ-tubulin* were used as housekeeping genes for normalization. The standard was made from PCR products amplified from cDNA using real time primer pairs for the housekeeping genes, and *DtFLC* (see supplementary data for sequences). These were combined and purified using a PCR purification kit (Qiagen) before diluting from 10^0^ to 10^−10^ for the standard curve. cDNA synthesized for each sample was used with the reaction mix iTaq™Universal SYBR^®^ Green Supermix (Bio-Rad Laboratories Ltd., UK). All real time PCR experiments were run on a CFX384 TouchTM Real-time PCR machine (Bio-Rad Laboratories Ltd., UK) using Bio-Rad CFX manager 3.0 software. Results were analyzed using Biogazelle qBase Plus software version 2.5 (http://www.biogazelle.com/qbaseplus).

## 3 Results

### 3.1 The effect of vernalization on flowering of *D.tenuifolia*

To investigate the vernalization response of wild rocket, both seed and young plants were tested for their response to different lengths of vernalization treatment at 4 °C. *D.tenuifolia* seed were subjected to a vernalization treatment of one, two or four weeks at 4 °C in the dark before being grown under ambient conditions (20 °C, 16 h photoperiod) in the glasshouse until they initiated bolting. The number of days to bolting was recorded starting from when the seed were taken out of the vernalization treatment (i.e. the time the seed spent in the vernalization treatment was not included). Control seed were not vernalized and were grown in ambient conditions for the duration of the experiment. There was no great effect on bolting time of the different lengths of vernalization treatment at 4 °C, except for a slight reduction of 4 days after one week of treatment which was not observed after two or four weeks of treatment (Fig. 1b). The effect of a 4 °C vernalization treatment on young four week old plants was also tested. The plants were subjected to two, four or eight weeks of cold treatment whilst a subset of control plants remained at constant ambient temperature throughout the experiment. There was no significant reduction in bolting time between the non-vernalized plants kept at ambient temperatures to those subjected to the different vernalization treatments (Fig. 1c). The plants which had been subjected to eight weeks of vernalization in fact bolted slightly later (approximately five days) than the control plants and the other vernalization treatments.

As wild rocket is a Mediterranean plant it may have a higher optimum vernalization temperature. To test this, a higher vernalization temperature of 10 °C was used for the vernalization treatment of both seed and young four week old plants. Overall the results mirror those subjected to a 4 °C vernalization as there was no great difference in bolting time between those kept under ambient conditions and the different vernalization treatments, although a small reduction in bolting time was observed following the six week treatment of seed (Supplementary data Fig. S1a). Vernalization of young plants at 10 °C did not reduce bolting time compared to non-vernalized plants kept at ambient temperature; indeed this treatment seemed to cause a delay in bolting (Supplementary data Fig. S1b).

### 3.2 Isolation of a rocket *FLC* gene

**Fig. 2:**
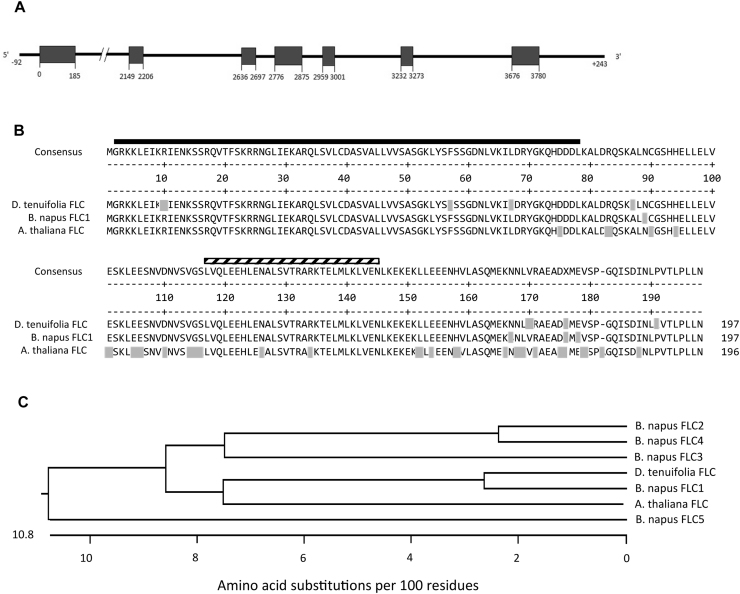
DtFLC gene and protein. A) Structure of DtFLC gene from D.tenuifolia. Boxes represent exons and lines represent introns and UTR. B) DtFLC protein alignment with B.napus FLC1 and A.thaliana FLC proteins. Grey shading identifies the residues that differ to the consensus sequence shown above. The black line denotes the MADS box domain and the hatched bar denotes the K-box motif. C) Phylogenetic tree showing the relationship between DtFLC, AtFLC and the five B.napus FLC proteins (1–5). DtFLC is most closely related to B.napus FLC1.

We isolated an *FLC* homologue from wild rocket to investigate whether expression of this gene changed in response to the vernalization treatment. PCR using primers designed to Arabidopsis and *Brassica FLC* genes gave fragments that had a sequence of an *FLC-like* gene, so specific RLM RACE (Invitrogen) primers were designed. This allowed the isolation of the full genomic sequence of the *DtFLC* gene revealing that it contained 7 exons and 6 introns (Fig. 2a). The intron sizes differ to that of Arabidopsis *FLC* but the exon sizes and placement are very similar (Michaels and Amasino, 1999).

The full length coding sequence for *DtFLC* has 94% identity to the *B.napus FLC1* and *Sinapis alba FLC* genes and 86% identity to *Arabidopsis thaliana FLC*. The translated protein sequence has 95% identity to *B.napus* FLC1 and *S.alba* FLC, and 84% identity to *A.thaliana* FLC, and contains a MADS box at the N-terminus and a K-box domain in the middle of the sequence (Fig. 2b). Phylogenetic comparison of DtFLC protein sequence with that of AtFLC and the five known FLC-like proteins of B.napus (FLC1-5) shows that DtFLC is most closely related to B.napus FLC1 (Fig. 2c).

### 3.3 Functional complementation of the Arabidopsis FRIGIDA (FRI)*^+^flc3* null mutant with *DtFLC*

**Fig. 3:**
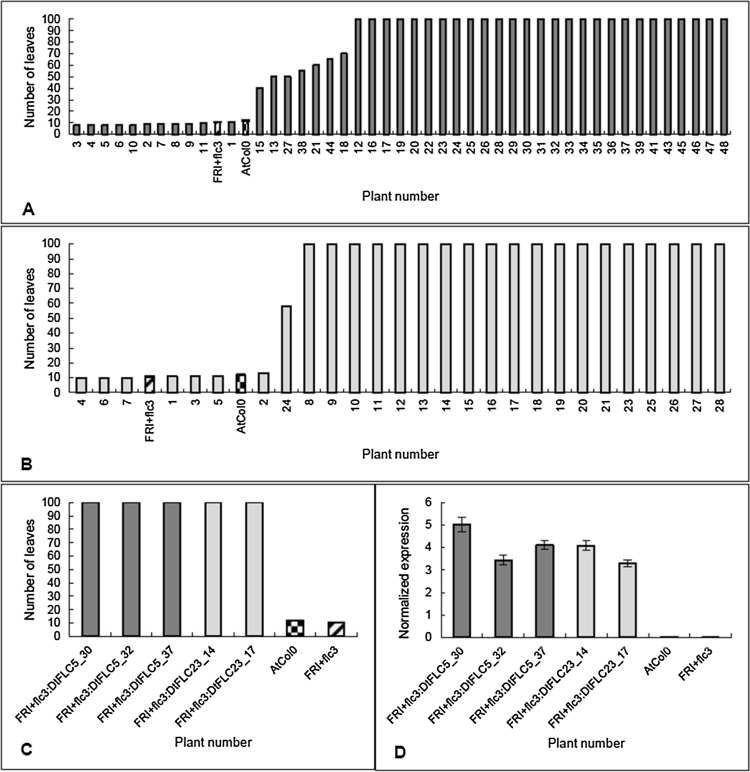
Flowering time and DtFLC expression data of FRI^+^flc3:DtFLC T_2_ lines. A) Flowering time data for line FRI^+^flc3:DtFLC 5. Rosette leaf number was recorded when bolt reached 1 cm. B) Flowering time data for line FRI^+^*flc3:DtFLC 23.* Checked bar shows mean number of rosette leaves at flowering (1 cm bolt) for *AtCol0* wild type (mean 11.8 ± 1.5 n = 24). Hatched bar shows mean number of leaves at flowering for FRI^+^*flc3* mutant (mean 10.7 ± 1.2 n = 21). Black bars at 100 leaves show plants that did not bolt before the end of the experiment. Error bars denote the standard error of the mean. Chi-squared tests assessing goodness of fit for data to an expected 3:1 late:early bolting phenotype show that lines FRI^+^*flc3:*DtFLC 5 and FRI^+^*flc3:DtFLC 23* were not significantly different to the 3:1 ratio (p < 0.05). C) Number of rosette leaves at 1 cm bolt of T_2_ plants from lines *FRI* *+* *flc3:DtFLC 5* (dark grey bars) and *FRI* *+* *flc3:DtFLC 23* (light grey bars) alongside WT *AtCol0* (checked bar) and *FRI* *+* *flc3* null mutant (hatched bar). Bars with 100 leaves did not bolt before the end of the experiment. Error bars denote standard error of the mean. D) Transgene expression analysis using real time PCR for T_2_ plants of lines *FRI* *+* *flc3:DtFLC 5* (dark grey bars) and *FRI* *+* *flc3:DtFLC 23* (light grey bars). Expression was normalized to the combined average expression of *Atβ-tubulin*, *AtActin2* and *AtTIP41*. Error bars denote standard error of three technical replicates.

The coding sequence for *DtFLC* was cloned into the vector pB2GW7 containing a 35S promoter and *Bar* gene which was transformed into Arabidopsis FRI*^+^flc3* null mutant plants by the floral dip method. The transformed seed were selected using BASTA and the T_1_ seed were grown and scored for bolting. The expression of the FLC floral repressor should result in delayed flowering/bolting and so T_1_ plants which were late bolting compared to the untransformed Arabidopsis *Col0* and FRI*^+^flc3* null mutant plants were harvested for seed. The T_2_ seed were sown out for the FRI^+^*flc3:DtFLC 5* and FRI^+^*flc3:DtFLC 23* lines and grown in a 16 h photoperiod to screen for the flowering phenotype. The T_2_ population for each line showed the expected segregation of 3:1 late:early flowering phenotype associated with the insertion of one copy of the transgene (Fig. 3a & b). A Chi-squared test was performed on the data and shows no significant difference to the 3:1 ratio. Overall the data suggest that *DtFLC* is able to complement the lack of function of *AtFLC* in FRI*^+^flc3* null mutant plants. RNA was extracted from a few T_2_ plants of the FRI^+^*flc3:DtFLC 5* and FRI^+^*flc3:DtFLC 23* lines to investigate the levels of expression of the transgene present in the transformed lines. Fig. 3c shows the number of leaves at flowering for the plants tested from both the FRI^+^*flc3:DtFLC 5* and FRI^+^*flc3:DtFLC 23* lines compared to the non-transformed *AtCol0* wild type and FRI*^+^flc3* mutant plants, and Fig. 3d shows the expression level of the *DtFLC* transgene in each of these plants. As expected there is no transgene expression detectable in the non-transformed *AtCol0* wild type and FRI*^+^flc3* mutant plants.

### 3.4 Expression of *DtFLC* during cold treatment of *D.tenuifolia*

**Fig. 4:**
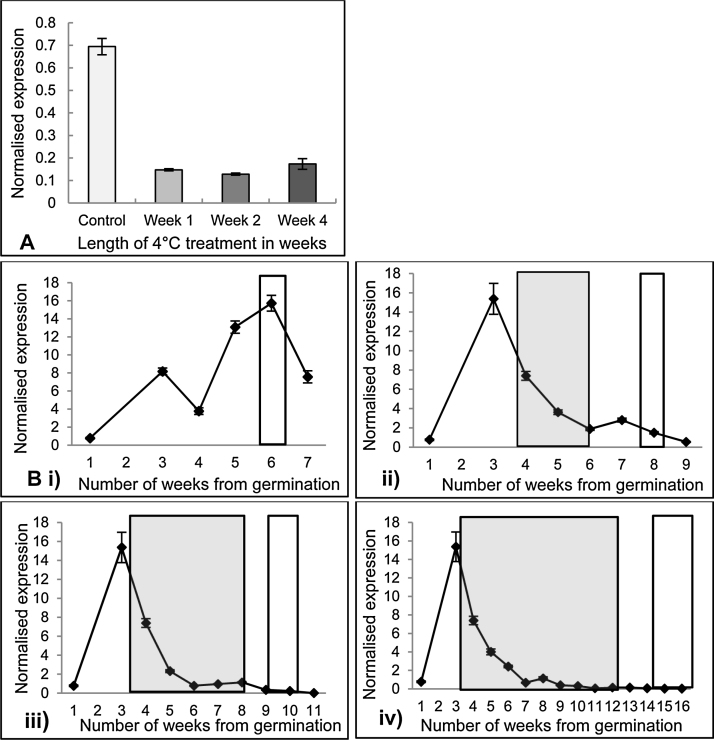
Effect of vernalization at 4 °C on DtFLC expression in D.tenuifolia. A) Expression of *DtFLC* where seed was subjected to vernalization at 4 °C for one, two or four weeks. Error bars denote the standard error of three biological replicates. B) Expression of *DtFLC* over development where 4 week old plants were subjected to vernalization at 4 °C for two, four or eight weeks. Change in levels of *DtFLC* expression over the course of the experiment under i) ambient conditions, ii) two weeks vernalization, iii) four weeks vernalization, iv) eight weeks vernalization. Error bars denote the standard error of three biological replicates. Range of bolting time of plants shown by white boxes and the respective 4 °C vernalization treatments shown by the grey boxes.

The isolation of *DtFLC* allowed the expression of the gene to be investigated in wild rocket following vernalization. In the 4 °C seed vernalization experiments, *DtFLC* expression was analyzed in the seed or seedlings collected at the end of each vernalization treatment (Fig. 4a) and the results revealed that those subjected to one week of ambient temperature conditions (control) had a much higher level of expression than in seed subjected to the vernalization treatments. This demonstrates that one week of cold treatment is sufficient to dramatically reduce the levels *DtFLC* expression. There is little change in the level of *DtFLC* expression during subsequent vernalization treatments indicating that once the expression level has been reduced in the first week the length of the vernalization treatment thereafter has no further effect on *DtFLC* expression.

The expression of *DtFLC* throughout plant development was investigated for each of the 4 °C vernalization treatments of four week old plants, and the non-vernalized control. In non-vernalized plants maintained in ambient conditions *DtFLC* expression increased up until three weeks after germination, fell slightly at four weeks, then increased again up until bolting and fell after the transition to flowering had occurred (Fig. 4bi). In plants that had been given the vernalization treatment, the pattern of *DtFLC* expression was the same as under the ambient conditions up to the point the vernalization treatment started (at four weeks after germination). When the vernalization treatment began, the levels of *DtFLC* fell to a low level and the level remained low, even when plants were returned to ambient conditions (Fig. 4bii, iii, iv). This shows that all of the cold treatments tested were effective in reducing the expression of *DtFLC* and maintaining it at a low level after vernalization.

### 3.5 Photoperiodic requirements of *D. tenuifolia*

**Fig. 5:**
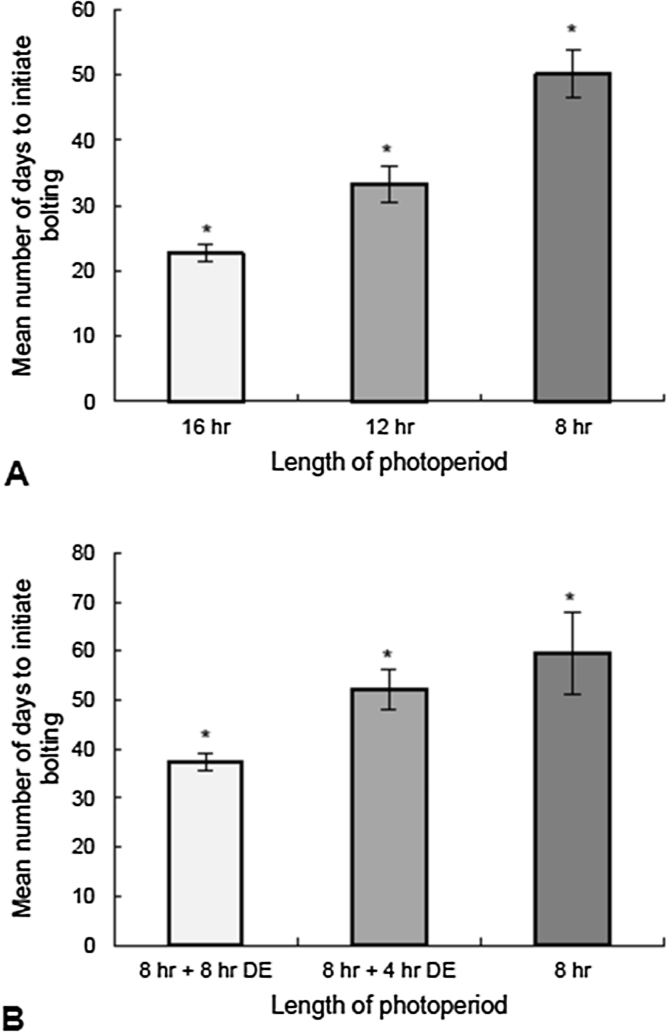
Effect on bolting time of varying photoperiod lengths in Diplotaxis tenuifolia plants. Mean number of days to initiate bolting for plants grown in 8 h, 12 h and 16 h photoperiods. A) Photoperiod experiment with light provided by fluorescent bulbs. Bars show mean ± SE (n = 9 (16 h + 12 h), n = 6 (8 h)). B) Photoperiod experiment with 8 h light provided by fluorescent bulbs and the 4 h or 8 h day extensions by incandescent bulbs. Bars show mean ± SE (n = 5 (8 h + 8 h DE, 8 h + 4 h DE), n = 6 (8 h)). Error bars denote the standard error of the mean. Ordinary one-way ANOVA showed a significant difference (*) between all photoperiod lengths on the initiation of bolting time (p < 0.05).

Three different photoperiods were used to investigate the effect of photoperiod on the number of days to initiate bolting in wild rocket. The experiment was carried out using fluorescent bulbs to provide photoperiods of 8 h, 12 h or 16 h (light levels 124 μmol.m^−2^.s^−1^). The data show that the plants respond differently to each photoperiod, with shorter photoperiods delaying bolting (Fig. 5a). There was a significant difference in the number of days to bolting between plants in each photoperiod (p < 0.05) (Fig. 5a). The experiment was repeated using fluorescent bulbs to provide a standard SD photoperiod of 8 h, with low levels of incandescent light (33 μmol m^−2^ s^−1^) used to provide the additional 4 and 8 h day extensions for the 12 h and 16 h photoperiods respectively. Low light levels were used for the day extension treatments to reduce photosynthesis levels therefore reducing any effects on flowering time that may be caused by photosynthesis/growth due to the additional 4 or 8 h light in the 12 h and 16 h photoperiodic treatments respectively. Incandescent bulbs rather than the fluorescent bulbs were used for the day extension treatments as these contain higher amounts of far-red light and are much more effective than fluorescent light for photoperiodic responses (Johnson et al., 1994). This experiment gave the same result as the first experiment in that shorter photoperiods caused a delay in bolting with there being a significant difference in the number of days to bolt between each photoperiod length (p < 0.05) (Fig. 5b).

## 4 Discussion

The vernalization and photoperiodic pathways are two key pathways involved in the perception of, and response to, environmental conditions. Different responses to vernalization and photoperiod are observed in different ecotypes of Arabidopsis (Strange et al., 2011) and in different varieties of crop plants such as wheat and oilseed rape (Bluemel et al., 2015, Srikanth and Schmid, 2011). Understanding the vernalization and photoperiodic requirements of wild rocket is important for commercial production in different parts of the world, and for breeding of new varieties with different flowering behaviours.

Prior to this set of experiments it was not known whether wild rocket has a vernalization requirement, and if so at which developmental stage and temperature the response is greatest. Our data shows that wild rocket does not respond to vernalization neither as seed nor as four week old plants, at either 4 °C or 10 °C. Vernalization of vernalization-responsive Arabidopsis accessions results in a large difference in flowering time between non-vernalized plants and vernalized plants, which flower much earlier (Sanchez-Bermejo et al., 2012), and if wild rocket has a vernalization requirement, we would expect to see similar large differences in bolting time. Although there is a small decrease in the mean number of days to bolt after vernalizing wild rocket seed for one week at 4 °C, it clearly doesn’t satisfy a putative vernalization requirement as longer periods of vernalization of two or four weeks at 4 °C resulted in a later bolting time (Fig. 1b). Similarly, whilst a small reduction in the number of days to bolt was observed following vernalization of seed at 10 °C for six weeks (Supplementary data Fig. S1a), a much greater reduction in bolting time would be expected if wild rocket possessed a vernalization response. Experiments with young four week old plants showed that there is no decrease in the mean number of days to bolt in response to vernalization treatments at either 4 °C or 10 °C (Figs. 1 c & S1b). In some cases, the vernalized plants take slightly longer to bolt than the non-vernalized controls grown under constant ambient conditions, which may reflect the need for a recovery period from the cold temperature which is likely to have slowed metabolic and physiological processes in the treated seed/young plants.

A homologue of the Arabidopsis *FLC* (*AtFLC*) gene was isolated from *D.tenuifolia* (*DtFLC*), the predicted protein sequence was found to have a higher identity to *B.napus* FLC1 than to AtFLC and other Brassica FLC proteins. *AtFLC* functions as a repressor of flowering and Kim et al. (2007) investigated how candidate *FLC* genes from Chinese cabbage (*B.rapa* L. ssp. Pekinensis) behaved when used to complement Arabidopsis *flc* mutants. They found that all *BrFLC* genes tested caused a late flowering phenotype showing that these genes were able to complement the *flc* mutant and were repressors of flowering. A similar approach was taken to investigate the function of *DtFLC*. The Arabidopsis *FRI^+^flc3* null mutant was transformed with the *DtFLC* coding sequence and late flowering lines were obtained in the T_1_ and T_2_ generations showing that *DtFLC* was able to complement the lack of functional *AtFLC* in the mutant *flc* lines. Analysis of transgene expression levels confirmed that the complemented lines were expressing the transgene (Fig. 3d). These results showed that *DtFLC* is a functional orthologue of *AtFLC* and enabled us to investigate the pattern of *DtFLC* gene expression during the various vernalization treatments used in our experiments.

When expression levels of *DtFLC* were examined in seed vernalized at 4 °C the results showed a large drop in expression during the first week of cold treatment and that levels remained low during additional weeks of vernalization (Fig. 4a), this reflects results obtained in Arabidopsis (Sheldon et al., 2000). The expression of *DtFLC* observed in the four week old plants subjected to vernalization at 4 °C showed a similar pattern in that one week of cold treatment was sufficient to cause a large reduction in expression levels. The levels of *DtFLC* were shown to remain low during longer periods of 4 °C cold, and were still low after the plants were returned to ambient conditions for the remainder of the experiment (Fig. 4b). Together, these results show that just one week of 4 °C cold treatment is effective in reducing the levels of *DtFLC* expression but that this does not have an effect on reducing the time to bolting.

The *DtFLC* expression data showed that the vernalization-induced reduction in expression levels of *DtFLC* is maintained even when plants are returned to ambient conditions (Fig. 4b). This suggests that wild rocket may have genes homologous to the Arabidopsis *VERNALIZATION INSENSITIVE 3*, *VERNALIZATION 1* and *VERNALIZATION 2* genes which reduce the expression of *FLC* and maintain the low levels after vernalization (Gendall et al., 2001, Levy et al., 2002, Sung and Amasino, 2004). However, as the reduction of *DtFLC* expression during and post-vernalization treatment does not affect bolting time in rocket, this suggests that the relationship between *FLC* expression and flowering in wild rocket may not be the same as in Arabidopsis. This may be due to some disruption of the flowering time pathway between *DtFLC* and the floral integrator genes meaning that the latter are unaffected by changes in *DtFLC* expression. It could also possibly be that in non-vernalized wild rocket *DtFLC* is expressed at levels which are insufficient to actively repress flowering and so the observed reduction in expression of *DtFLC* during vernalization would not have any significant effect on flowering. This would be similar to Arabidopsis ecotypes *Ler*, *Da* and *Shakhdara* which have a low expression of *AtFLC* but flower rapidly and are classified as summer annuals. It was found that the *AtFLC* gene in *Ler*, *Da* and *Shakhdara* did not contain any mutations in the coding sequence compared to *Col0*, but the low expression was caused by mutations in intron 1 (*Ler*) or the 5′ UTR (*Da* and *Shakhdara*) sequences (Michaels et al., 2003). Overall the results demonstrate that rocket does not have a vernalization requirement as the cold treatments (either 4 °C or 10 °C) did not reduce the time the plants took to bolt compared to non-vernalized plants. This could be due to its origins in the Mediterranean where a vernalization response would be a disadvantage as has been found in *B.napus* varieties grown in Mediterranean climates where the lack of a vernalization requirement is important for its early flowering phenotype (Dahanayake and Galwey, 1999). Other studies in *B.rapa* have also shown that vernalization isn’t a requirement for plants bred in warmer climates (Franks et al., 2015).

Studies in Arabidopsis have explored the pattern of ecotype distribution and flowering time response particularly in relation to photoperiod and vernalization responses (Caicedo et al., 2004, Lempe et al., 2005, Stinchcombe et al., 2004). They linked the different degrees of response to differences in the activity of *FRI* (which promotes FLC expression) and *FLC*. Further investigation, for example the identification of a *FRI* homologue in rocket, may provide a greater understanding of vernalization responses in Mediterranean crops.

The photoperiodic requirement of wild rocket has not been properly defined and so experiments were set up to test the response of *D.tenuifoila* to different photoperiods. Plants were able to flower in LD (16 h), intermediate (12 h) and SD (8 h) photoperiods but flowered much more rapidly in longer photoperiods (Fig. 5), this shows that wild rocket is a facultative LD plant. Results of photoperiodic experiments using day extensions of low levels of incandescent light day to minimise the effects of additional photosynthesis on the control of bolting in the longer photoperiods were consistent with the first experiment, confirming the conclusion that rocket is a facultative LD plant.

## 5 Conclusions

A rocket homologue of *FLOWERING LOCUS C* (*DtFLC*) has been isolated and shown to functionally complement the *Arabidopsis* FRI*^+^flc3* null mutant. The expression of *DtFLC* is significantly reduced by one week of cold, however vernalization treatments of up to eight weeks had no significant effect on bolting time of rocket demonstrating that rocket does not have a vernalization requirement. The flowering response of rocket to different photoperiods show that flowering is promoted by LD and that rocket is a facultative long day plant.

## Author contributions

J.T., A.M. and S.J. designed the experiments, J.T. performed the experiments, J.T. and S.J wrote the manuscript, A.M., S.J., S.K. and Y.H. supervised the study and revised the manuscript.

## Conflict of interest

The authors declare that there are no conflicts of interest.
