# Supplementary material for: FLC expression is down-regulated by cold treatment in Diplotaxis tenuifolia (wild rocket), but flowering time is unaffected
Source: J Plant Physiol. 2017 Jul;214:7–15. doi: 10.1016/j.jplph.2017.03.015 (PMC5477103; doi:10.1016/j.jplph.2017.03.015)
Supplement: Supplementary file 1 [file mmc1.docx]

**ELECTRONIC SUPPORTING INFORMATION**

***FLC* expression is down-regulated by cold treatment in *Diplotaxis tenuifolia* (wild rocket), but flowering time is unaffected.**

Jemma L Taylor, Andrea Massiah, Sue Kennedy, Yiguo Hong, and Stephen Jackson

**Primers used throughout the experiment**

**Table S1 – Primer sequences used for *DtFLC* isolation and complementation experiments**

1. Degenerate primers used for initial *DtFLC* isolation

| FLC_1_F | TACAGCTTCTCCTCCGGCGATAAC | Pair annealing temperature: 61°C |
| --- | --- | --- |
| FLC_1_R | CAGCTTCGGCTCCCACAAGATTAT |  |
| FLC_2_F | TCAAGATCCTTGATCGATATGGGA | Pair annealing temperature: 58°C |
| FLC_2_R | GCTCTAGTTACGGAGAGGGCAGTC |  |
| FLC_3_F | GTGGATAGCAAGCTTGTGGAATCA | Pair annealing temperature: 58°C |
| FLC_3_R | TAGTGGGAGAGTTACCGGAAGAT |  |

1. Primers used for RACE PCR (according to manufacturer’s instructions)

| GSP_5’_FLC1-3_R (RACE) | TCCAACTGAACGAGGGAACCGACAC |
| --- | --- |
| GSP_5’_FLC2_R (RACE) | CTGCCCATGAAAAAGGGACAGAAG |
| GSP_5’_FLC2_nest_R (RACE) | GGGACAGAAGAAGCGGTAAAAGACAAGG |
| GSP_3’_FLC1-3_F (RACE) | CCCTCTCCGTAACAAGAGCTAGGAA |
| GSP_3’_FLC1-3_nest_F (RACE) | CAATCTTCCGGTAACCTCCCACTGAA |
| GSP_3'_FLC1-3_nest_2F (RACE) | GCTGGAAGAGGAGAACCATGTTTTGG |
| GSP_3’_FLC2_F (RACE) | GCCCTTGTCTTTTACCGCTTCTTC |
| GSP_3’_FLC2_nest_F (RACE) | GCAGCAAGCTTGTGGGATCAAATGTC |

1. Primers used for full length gDNA isolation

| DtFLC_5'_UTR_1_F | TGGTAACCCAACCTCAAGATCAAA | Pair annealing temperature: 60°C |
| --- | --- | --- |
| DtFLC_3'_UTR_1_R | GGATGCTTAAGGAAGCTCGAAGTA |  |

1. Additional DtFLC primers used in sequencing reactions

| DtFLC_exon1_F | CTTCTCCAAACGACGCAATGGTCTC |
| --- | --- |
| DtFLC_exon2_F | GATCGATATGGAAAGCAACATGATG |
| DtFLC_exon2_R | CTTTAAGATCATCATCATGTTGCTTTC |
| DtFLC_exon3_F | CGTCAGTCAAAACCTCTGAACTGTG |
| DtFLC_exon3_R | AGTTCCAGTAGCTCGTGGTGTGAACCA |
| DtFLC_exon4_F | GCTTGAGGAGTCAAACGTCGATAATG |
| DtFLC_exon4_R | CATTAGTTCTGTCTTCCTAGCTCTT |
| DtFLC_exon5_F | AGAAAAGGAGAAATTGCTGGAAGAGGAG |
| DtFLC_intron1_in1_F | TTCTTGTTGTCTCCGCCTCT |
| DtFLC_intron1_in1_R | TCCCATAGTTGCACACTGGT |
| DtFLC_intron1_in2_F | TTTACCCCACACCTAATGAACAC |
| DtFLC_intron_1_R | CCATGTCCATAGAAAAACAGTAGCA |
| DtFLC_intron_6_F | AGCCTTTAGGCTCCTAGAGAAAGC |

1. GATEWAY primers

| DtFLC_full_comp_att_1_F | GGACAAGTTTGTACAAAAAAGCAGGCTATGGGGAGGAAGAAACTTGA | Annealing temperature: 65°C |
| --- | --- | --- |
| DtFLC_full_comp_att_1_R | GGGGACCACTTTGTACAAGAAAGCTGGGTCTAATTAAGCAGTGGGAGTGTTAC |  |

**Table S2 – Primer sequences used for *DtFLC* real time RT-PCR experiments**

1. Primers for rocket real time PCR

| **Housekeeping gene primers for normalization** | DtTIP41_RT_1_F | CCATACTGTGGTAGCGGAGTTGTT | Annealing temperature used for all pairs was 59°C |
| --- | --- | --- | --- |
|  | DtTIP41_RT_1_R | AGACTAGCTTCCCCCTCAGGACTT |  |
|  | DtCACS_RT_1_F | CCTAGTGGTCGGAGAAAAGAGGAA |  |
|  | DtCACS_RT_1_R | GTGCCGTCAATCTCAGAAGTCAGT |  |
|  | Dtα-tubulin_RT_1_F | GTTCCCAAAGACGTTAATGCTG |  |
|  | Dtα-tubulin _RT_1_R | TTGGAGGTTGGTAGTTGATTCC |  |
| **Gene specific primers** | DtFLC_RT_1_F | CGTAACAAGAGCTAGGAAGACAGA |  |
|  | DtFLC_RT_1_R | CGCAAGATTATTCTTCTCCATC |  |

1. Primers used for Arabidopsis complementation of *DtFLC* T_2_ real time PCR

| **Arabidopsis housekeeping gene primers for normalization** | At_ACTIN2_RT_1_F | TGTCGCCATCCAAGCTGTTCT | Annealing temperature used for all pairs was 59°C |
| --- | --- | --- | --- |
|  | At_ACTIN2_RT_1_R | GTGAGACACACCATCACCAGAAT |  |
|  | At_TIP41_RT_1_F | ATGGTGTGCTTATGAGATTGAGAG |  |
|  | At_TIP41_RT_1_R | ATACCCTTTCGCAGATAGAGACTG |  |
|  | AtTUBBY_RT_1_F | TGGCAAGATGAGCACAAAAG |  |
|  | AtTUBBY_RT_1_R | AGACCTCGGGGAGCTATG |  |
| **Transgene specific primers** | DtFLC_RT_2_F | GGTTCCCTCGTTCAGTTGGAA |  |
|  | DtFLC_RT_2_R | CTCCATCTGGCTAGCCAAAAC |  |

**Effects of 10°C vernalization treatments on *D. tenuifolia***

*

Figure S1 - Effect of a 10°C vernalization treatment on seed or young plants on the bolting time of *D.tenuifolia*

A) Mean number of days to bolt of plants grown from seed subjected to a vernalization treatment of 10°C for two, four and six weeks. Bars show mean ± SE (n=6). B) Mean number of days to bolt of young four week old plants subjected to a vernalization treatment of 10°C for two, four and six weeks. Bars show mean ± SE (n=5 (0 weeks), n=6 (2, 4 and 6 weeks)). Student’s *t*-test was used to compare the number of days to bolt of each vernalization treatment (2, 4 and 6 weeks) against the control ambient conditions (0 weeks). *Statistical significance of p<0.0001

Methods for the 10°C vernalization experiment:

Seed vernalization: Rocket seed was sown onto damp paper towels and covered with aluminum foil. This was then placed into a controlled environment cabinet (MLR-352, Panasonic Co. Ltd) at 10°C in the dark to vernalize. After two weeks, all seed had germinated, so were transplanted onto Arabidopsis mix soil (Levington F2S:sand:vermiculite fine grade 6:1:1) in p24s and returned to 10°C but with low light levels (36 µmol.m^-2^.s^-1^) as done in Nordborg and Bergelson (1999) and Lin et al. (2005), so that plants would stay healthy but not grow very much. The total length of the vernalization treatments were two, four or six weeks at 10°C. The plants were transferred to a controlled environment cabinet (MLR-352, Panasonic Co. Ltd) with 22°C and 16 h photoperiod at the end of each treatment. At 5-6 true leaves, plants were transferred to 5 inch pots of M2 soil (Levington) and grown until initiation and bolting. Controls were kept continuously at ambient temperature (22°C and a 16 h photoperiod) in a controlled environment cabinet (MLR-352, Panasonic Co. Ltd). Germination of the control seedlings occurred after one week, so were transferred onto soil (Levington F2S:sand:vermiculite fine grade 6:1:1) in p24s and placed back into the cabinet at 22°C with 16 h photoperiod until initiation and bolting occurred. Plant material was harvested every week with RNA being extracted from seeds at the start of the experiment and week one and germinated seedlings destructively sampled for weeks two to six.

Plant vernalization: Seed was sown directly into 5 inch pots of Arabidopsis mix soil and placed into a controlled environment cabinet (Versatile plant growth MLR-352, Panasonic Co. Ltd) at 22°C with a 16 hour photoperiod. At four weeks after sowing, plants had six to seven true leaves and were moved to a controlled environment cabinet (Panasonic) at 10°C with a 16 hour photoperiod to begin the vernalization treatment. Treatments were for two weeks, four weeks and six weeks before returning the plants to ambient conditions in a controlled environment cabinet at 22°C with a 16 hour photoperiod until initiation and bolting. For plants grown as a control in ambient conditions, seeds were kept in a controlled environment cabinet (Versatile plant growth MLR-352, Panasonic Co. Ltd) at 22°C with a 16 hour photoperiod throughout the experiment. Initiation and bolting dates were recorded, and plant material collected throughout the experiment at weekly intervals at ZT9.
